# Roles and Mechanisms of Histone Deacetylases in Plant Abiotic Stress Responses

**DOI:** 10.3390/antiox15080960

**Published:** 2026-07-31

**Authors:** Enyang Lv, Panfeng Yao, Jiangyuan Qin, Zigang Liu, Yan Fang, Zefeng Wu, Guoqiang Zheng, Junmei Cui, Jiaping Wei

**Affiliations:** State Key Laboratory of Aridland Crop Science, Gansu Agricultural University, Lanzhou 730070, China

**Keywords:** HDACs, histone deacetylation, non-histone acetylation, abiotic stress, chromatin remodeling, oxidative stress, plant stress adaptation, epigenetic regulation

## Abstract

Histone deacetylases (HDACs) are key epigenetic enzymes governing lysine deacetylation. This modification is tightly coupled to cellular redox homeostasis and antioxidant signaling in plants. Plant HDACs are grouped into three subfamilies: RPD3/HDA1, SIR2, and plant-specific HD2. HDACs target both histone residues (e.g., H3K9 and H4K5) and a broad set of non-histone substrates (e.g., transcription factors and metabolic enzymes). Via coordinated chromatin remodeling and non-histone protein modification, HDACs integrate phytohormone signals, reactive oxygen species (ROS) bursts and NAD^+^ metabolic fluctuations to orchestrate plant abiotic stress responses, balancing antioxidant defense, redox equilibrium and normal growth. This review systematically sorts the divergent stress-response traits, substrate preferences and bidirectional regulatory logic of the three HDAC subfamilies; integrates chromatin-dependent and transcription factor-centered transcriptional branches; and summarizes crosstalk rules between HDAC-mediated deacetylation and other epigenetic marks. We further hierarchically clarify current research bottlenecks spanning basic mechanism dissection, multi-crop validation and field breeding transformation and propose targeted stratified research directions. We further construct a complete regulatory cascade linking environmental stimuli, ROS/ABA/NAD^+^ signals, HDAC activity and downstream antioxidant/stress gene expression, filling gaps in previous reviews that overlook redox-dependent HDAC functions. This mechanistic framework delivers integrated epigenetic and redox theoretical references for breeding stress-tolerant crops with reinforced antioxidant capacity.

## 1. Introduction

Abiotic stresses, including extreme temperature, soil salinity, drought, oxidative stress and heavy metal exposure, trigger massive reactive oxygen species (ROS) accumulation, disrupt cellular redox balance and impair enzyme activity, collectively reducing global crop yields by over 50% annually [1]. Worse still, climate change is increasing the frequency and intensity of these stressors. The Food and Agriculture Organization (FAO) has predicted that climate-related disasters will reduce crop yields by an additional 15–30% by 2050. This threatens food security, particularly in vulnerable regions. Unlike animals, plants are sessile organisms, lacking the ability to evade adverse environments. They have therefore evolved intricate adaptive mechanisms to perceive, transduce, and respond to abiotic stress signals. Among these, epigenetic reprogramming has emerged as a pivotal strategy, enabling plants to dynamically modulate the expression of stress-responsive genes without altering DNA sequence [2,3].

Histone acetylation is one of the earliest-confirmed epigenetic modifications involved in plant abiotic stress [4]. This dynamic and reversible modification is based on two enzyme families with antagonistic functions. Histone acetyltransferases (HATs) add acetyl groups to lysine residues, whereas histone deacetylases (HDACs) remove these acetyl modifications [5]. Acetylation relaxes chromatin to enable transcriptional activation, whereas histone deacetyla-tion drives chromatin compaction and transcriptional repression [6,7]. However, accumulating evidence demonstrates that HDACs also target a substantial number of non-histone proteins, with over 30% of their targets comprising redox-sensitive transcription factors, glycolytic enzymes, and antioxidant stress-signaling molecules [8,9,10]. This expanded substrate suggests that HDACs are versatile regulators that integrate chromatin dynamics with cellular signaling to regulate plant growth, development, and stress resilience [11,12,13].

However, the substrate specificity and catalytic efficacy of HDACs are strictly dependent on the stoichiometric availability of cofactors, namely Zn^2+^ (for RPD3/HDA1 classes) and NAD^+^ (for SIR2/sirtuins). Critically, abiotic-stress-triggered respiratory bursts and energy deficits inevitably provoke dramatic fluctuations in the intracellular NAD^+^/NADH ratio and the accumulation of reactive oxygen species (ROS) such as H_2_O_2_ and O2^−^. These fluctuations directly influence HDAC catalytic efficiency by altering substrate accessibility and cofactor availability. Concurrently, ROS-mediated oxidative post-translational modifications (e.g., cysteine sulfenylation) can conversely modulate the protein stability and nucleocytoplasmic shuttling of HDACs themselves. Hence, HDACs are far from passive epigenetic writers executing transcriptional repression; rather, they function as active sentinels that perceive cellular redox perturbations and orchestrate nuclear transcriptional reprogramming in real time.

Despite the growing number of HDAC targets identified under various abiotic stresses, most existing reviews and primary studies have treated epigenetic regulation and oxidative stress responses as largely separate topics. A systematic framework that links HDAC deacetylation dynamics with real-time ROS signaling, NAD^+^/NADH metabolic flux, and the buffering capacity of the antioxidant enzyme network remains conspicuously lacking. In particular, the regulatory roles of NAD^+^-dependent sirtuins in plants are still fragmentarily understood, and the feedback regulation of HDAC activity by stress-induced oxidative modifications of cysteine residues is rarely discussed in an integrated redox-epigenetic context. This fragmented view hinders the translation of HDAC knowledge into strategies for enhancing endogenous antioxidant capacity in crops.

In this review, we focus on the interdependency between HDAC deacetylation, ROS signaling, NAD^+^ metabolism and plant antioxidant systems and propose tiered experimental priorities for future redox-epigenetic research. We systematically summarize plant HDAC classification, structural biochemistry, and multilayered regulatory molecular mechanisms in response to abiotic stress, with sustained emphasis on oxidative stress responses and redox homeostasis. This work provides a unified epigenetic-redox framework to guide crop stress resistance breeding focused on enhancing endogenous antioxidant capacity.

## 2. The Histone Deacetylase Family in Plants

### 2.1. Discovery and Genome-Wide Identification of Plant HDACs

The study of histone deacetylase (HDAC) began with the observation of histone acetylation dynamics. In 1964, Allfrey and his colleagues first discovered histone acetylation and proposed the existence of enzymes capable of removing histone acetyl groups [4]. This insight laid the foundation for HDAC research. However, the first HDAC (HDAC1) was not identified from human cells until 1996 [14], marking a key milestone. In the same year, Brosch et al. isolated and purified the first plant HDAC, maize HDA1 [15]. These discoveries mark a transition of HDAC research from theoretical speculation to the era of molecular genetic interrogation.

Advances in sequencing technology have accelerated the identification of HDAC genes. Up to now, the HDAC family has been identified in more than 30 plant species (Table 1). However, the number of HDACs varies significantly among different species. For instance, *Hibiscus cannabinus* has six HDACs, while wheat (*Triticum aestivum*) has 49 HDACs [16,17]. This variation likely reflects evolutionary history, genome size, and ecological adaptations, indicating the presence of gene expansion or contraction events in different plant species.

### 2.2. Classification, Structural Features and Catalytic Mechanisms

**Figure 1:**
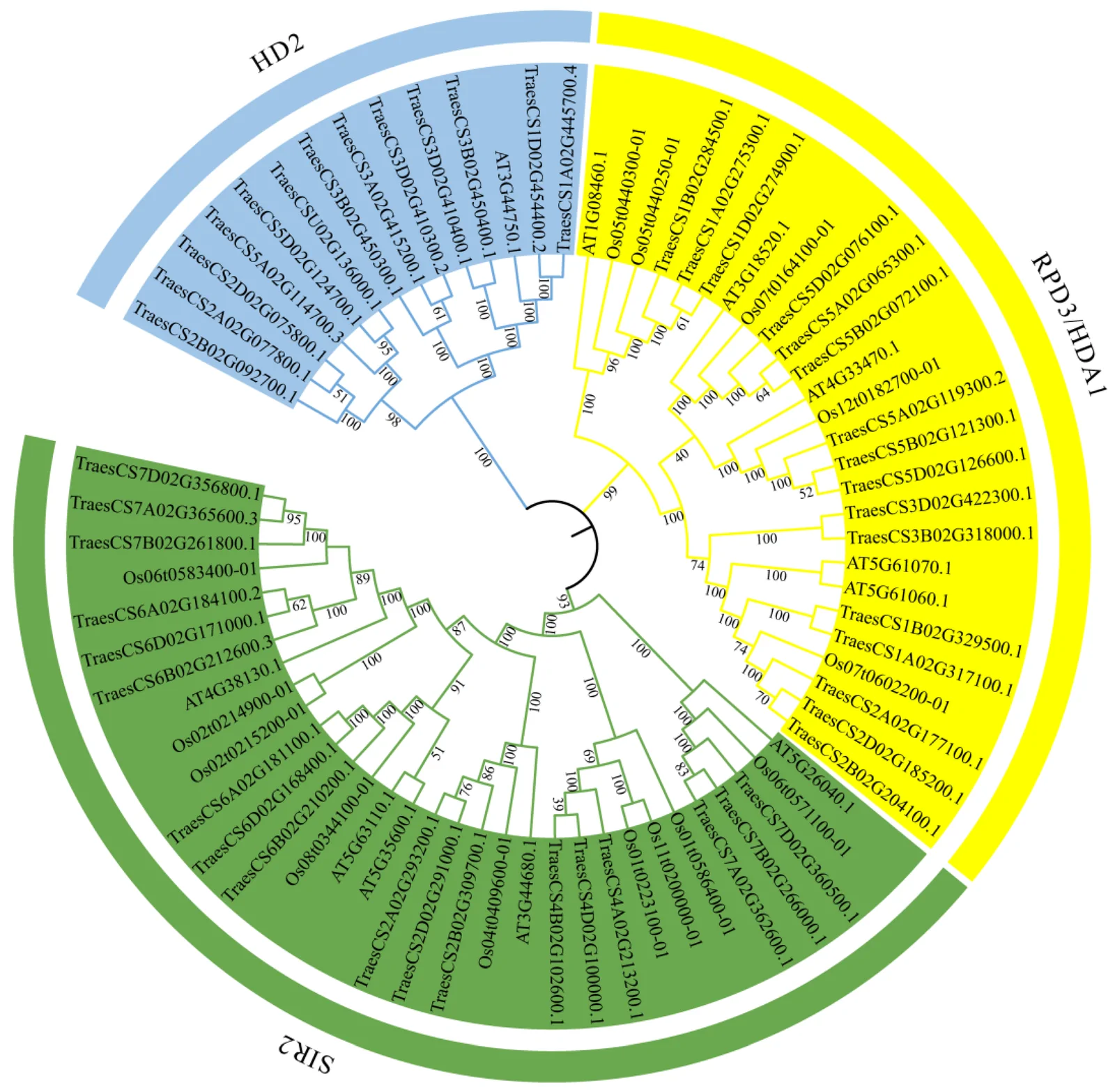
Phylogenetic analysis of plant histone deacetylases (HDACs). A phylogenetic tree of HDAC family members from rice, wheat, and *Arabidopsis* was constructed. The HDAC members were grouped into the RPD3/HDA1 (yellow), SIR2 (green), and HD2 (blue) subfamilies. All protein sequences were aligned via the ClustalW algorithm integrated in MEGA software version 7. The phylogenetic tree was constructed using the Neighbor-Joining (NJ) method with amino acid sequences as the substitution type, and branch support was assessed by bootstrap analysis with 1000 replicates. Numbers on the branches represent bootstrap support values. The p-distance model was adopted to calculate genetic distances; uniform evolutionary rates across all sites and homogeneous substitution patterns among lineages were set. Gaps and missing data were processed with partial deletion, with a site coverage cutoff of 50%, while other parameters were kept as software default values. The Newick format tree file exported from MEGA was imported into the iTOL online platform for tree visualization, branch coloring and esthetic optimization.

Based on sequence homology, domains, and catalytic mechanisms, plant HDACs are classified into three subfamilies (Table 1 and Figure 1): RPD3/HDA1 (Reduced Potassium Dependence 3/Histone Deacetylase 1), SIR2 (Silent Information Regulator 2), and HD2 (Histone Deacetylase 2) [33].

#### 2.2.1. RPD3/HDA1 Subfamily

The RPD3/HDA1 subfamily represents one of the most conserved and well-characterized zinc-dependent HDACs in eukaryotes. This family is named after two classic prototype proteins identified in *Saccharomyces cerevisiae*, namely RPD3 and HDA1. Conserved catalytic domains are commonly found across all RPD3/HDA1 members, which require zinc ions as core cofactors to drive deacetylation. Correspondingly, common HDAC inhibitors such as trichostatin A (TSA) and suberoylanilide hydroxamic acid (SAHA) can specifically suppress the catalytic activity of these proteins. According to evolutionary traits and structural features, this family is grouped into three subclasses, namely Class I (RPD3 subfamily), Class II (HDA1 subfamily) and Class IV. Class I proteins mainly reside in the nucleus, where they facilitate transcriptional complex assembly and mediate gene silencing. In contrast, Class II proteins are capable of nucleocytoplasmic shuttling, exerting separate biological functions in the nucleus and cytoplasm. In contrast, Class IV members show relatively low evolutionary conservation and possess specific functional characteristics.

#### 2.2.2. SIR2 Subfamily

The SIR2 subfamily relies entirely on NAD^+^ to sustain its catalytic activity, setting it apart from zinc-dependent RPD3/HDA1 HDACs. The term SIR2 originates from Silent Information Regulator 2, a gene first characterized in *S. cerevisiae*. Early studies proved that the yeast SIR2 protein maintains transcriptional silencing at telomeric regions and mating-type loci. Every member of this family bears a conserved Sirtuin catalytic domain. NAD^+^ acts as an indispensable co-substrate during deacetylation, and nicotinamide produced from this catalytic reaction can feedback-inhibit enzymatic activity. Unlike RPD3/HDA1 proteins, SIR2 members barely respond to TSA or SAHA. Certain SIR2 variants even exhibit mono-ADP-ribosyltransferase activity besides deacetylase function. Phylogenetic analyses divide eukaryotic SIR2 proteins into distinct subgroups based on sequence divergence and subcellular distribution. Mammals encode seven paralogs named SIRT1 to SIRT7, which are distributed in the nucleus, cytoplasm or mitochondria to execute compartment-specific tasks. Plants carry fewer SIR2 homologs, which largely modulate stress tolerance and developmental progression, while yeast only harbors a single SIR2 gene. This NAD^+^ dependency places plant SIR2s at the core of redox-epigenetic crosstalk: abiotic stress-triggered ROS accumulation disturbs NAD^+^/NADH ratios, directly switching Sirtuin catalytic output to modulate antioxidant enzyme transcription.

#### 2.2.3. Plant-Specific HD2 Subfamily

The HD2 family, also designated HDT or HD-tuin, is a plant-specific subclass of HDACs. This family got its name from the second type of histone deacetylase complex initially isolated from maize chromatin extracts and is hence abbreviated as HD2. Commonly, HDT serves as its standardized systematic abbreviation in phylogenetic research. HD2s share almost no sequence homology with RPD3/HDA1 or SIR2 superfamilies, featuring a tripartite structural layout. At the N-terminus lies a conserved nucleoplasmin (NPL) domain marked by the universal pentapeptide MEFWG. Two core residues within this domain, histidine and aspartate, are indispensable to maintaining deacetylation activity. The central segment consists of a highly variable acidic stretch that enables physical binding to histone H3 and H4 as histone chaperones. Most HD2 proteins carry a C-terminal cysteine-rich zinc finger domain, which facilitates DNA or protein binding. Unlike RPD3/HDA1 or SIR2, HD2 enzymatic activity is dependent neither on Zn^2+^ nor on NAD^+^. Conventional HDAC inhibitors such as TSA, SAHA and nicotinamide fail to block their enzymatic activity. Most HD2 isoforms accumulate within the nucleolus, while a small subset shuttle between nucleus and cytoplasm; phosphorylation modification widely controls their catalytic potency in vivo. Four HD2 paralogs (HD2A/HDT1, HD2B/HDT2, HD2C/HDT3, HD2D/HDT4) have been characterized in *A. thaliana*, and copy numbers of HD2 genes vary substantially across monocot and dicot crops.

#### 2.2.4. Functional Divergence of Three HDAC Subfamilies

Distinct sequence features, cofactor dependencies and inhibitor responses divide plant HDACs into three functionally specialized subfamilies with clear labor division and partial functional overlap. The widely distributed RPD3/HDA1 subfamily participates in multiple fundamental biological processes, including plant growth and development, stress tolerance, cell differentiation and genome stability maintenance. Differently, the SIR2 subfamily functions as a key cellular energy sensor, which integrates intracellular metabolic status with critical biological events such as cellular senescence, DNA damage repair and oxidative stress resistance. As a unique plant-specific HDAC subfamily, HD2 members exert predominant regulatory effects on seed dormancy, leaf development and floral transition. In addition, these proteins also enable plants to cope with diverse adverse environments, including drought, salt stress and pathogenic invasion.

Despite well-established sequence-based classification frameworks, current comparative research remains superficial, with few parallel assays systematically contrasting how the three subfamilies differentially respond to fluctuating ROS and NAD^+^ levels under oxidative stress, leaving a critical gap in redox-epigenetic mechanistic understanding.

## 3. Functions of HDACs in Abiotic Stress Response

### 3.1. Stress-Dependent Transcriptional Reprogramming of HDAC Genes

HDACs serve as core epigenetic regulators that participate extensively in plant stress adaptation. Notably, their transcriptional abundance is dynamically altered by external environmental stimuli, and such expression reprogramming acts as the initial molecular event for HDACs to integrate upstream stress signals into intracellular regulatory networks. Accumulating transcriptomic and fluorescence quantitative data evidence has revealed that three HDAC subfamilies exhibit conserved yet divergent transcriptional regulatory patterns in response to abiotic stresses. Based on the family classification of HDACs, this section further summarizes and compares the distinct stress-responsive expression and regulatory characteristics of the three HDAC subfamilies under abiotic stress, focusing on their subfamily-specific expression rules.

The HD2 subfamily presents a typical negative regulatory expression pattern in response to salt stress and ABA treatment. In *A. thaliana*, all four HD2 paralogs (*AtHD2A* to *AtHD2D*) are transcriptionally suppressed under abscisic acid (ABA) and salt treatments [11]. This expression pattern is also observed in *Camellia sinensis*, where the HD2 homologous gene *CsHDC2* exhibits identical downregulation upon ABA and NaCl exposure, suggesting conserved evolutionary functions of HD2 proteins in salt and ABA signaling pathways [30]. Similarly, *PtHDT902* from poplar is repressed by salt, mannitol, and ABA across multiple tissues [35]. However, the expression of *PmHDT1* in *Prunus mume* is induced by cold stress [22]. Taken together, HD2 subfamily genes are generally negatively regulated at the transcriptional level under salt and ABA stress, forming a stable stress-responsive expression regulatory mode.

In contrast to the negative regulation of the HD2 subfamily, the RPD3/HDA1 subfamily mainly shows positive transcriptional regulation in response to abiotic stress and stress-related phytohormone stimuli. In *Dendrobium officinale*, the RPD3/HDA1 family member *DoHDA10* harbors drought-responsive *cis*-acting elements within its promoter region. Quantitative PCR assays revealed that drought, salicylic acid (SA) and methyl jasmonate (MeJA) all significantly stimulate the transcription of *DoHDA10* [31]. Given that SA and MeJA function as core signaling phytohormones governing plant responses to abiotic stress, we hypothesize that these two compounds may act as upstream signals to trigger transcriptional activation of *DoHDA10*, thereby co-modulating plant adaptation to drought conditions. This phytohormone-mediated transcriptional activation of RPD3/HDA1 members constitutes a crucial positive regulatory mechanism for plant drought stress response. In addition to drought stress, cold stress also triggers widespread transcriptional activation of RPD3/HDA1 genes across plant species, including *AtHDA6* in *Arabidopsis*; *SbHDA12* in *Sorghum bicolor*; and *PmHDA1*, *PmHDA6* and *PmHDA14* in *P. mume* [21,22,36]. Notably, *AtHDA9* in *Arabidopsis* and *RsHDA9* in radish are upregulated by heat stress [34,37].

For the SIR2 subfamily, most genes are significantly upregulated under cold and drought stress. *SbSRT1* in *S. bicolor* and PmSRT1/PmSRT2 in *P. mume* are strongly induced by cold stress, contributing to plant cold acclimation [21,22]. Notably, all three HDAC subfamilies (HD2, RPD3/HDA1, SIR2) are uniformly upregulated under cold stress [21,22,36], which enables their synergistic regulation of plant cold adaptation. In *Brassica napus*, SIR2 subfamily genes are obviously upregulated at the early stage of drought treatment to participate in drought adaptation [38]. *AtSRT2* exhibits high expression in seeds. Its transcriptional level is induced under salt stress, and the gene further regulates salt tolerance and seed germination [39].

Overall, the three HDAC subfamilies present stress-specific transcriptional signatures coordinated by ABA, ROS and NAD^+^ signals, yet time-series comparative transcriptomics under oxidative stress remain limited. The HD2 subfamily is downregulated under salt and ABA stresses; the RPD3/HDA1 subfamily is upregulated under drought, heat, SA and MeJA treatments; the SIR2 subfamily is upregulated under cold, salt and drought stresses. In particular, all three subfamilies share a conserved upregulated response to cold stress, forming a unified regulatory mode for plant cold acclimation. These distinct and synergistic expression regulatory patterns collectively enable HDACs to participate in plant abiotic stress adaptation through diversified molecular mechanisms, laying a solid transcriptional basis for their versatile biological functions.

### 3.2. Histone Lysine Deacetylation Regulates Abiotic Stress

HDACs modulate downstream stress signaling cascades mainly through removing acetyl groups from specific lysine residues on histone tails. This epigenetic modification remodels chromatin accessibility and further controls the transcription of stress-responsive genes. Such chromatin-dependent regulation enables plants to precisely adjust stress-related transcriptional programs. In addition, individual HDAC subfamilies target different histone modification sites, thereby exerting either positive or negative regulatory effects on plant stress resistance (Table 2).

For the RPD3/HDA1 subfamily, its members modulate abiotic stress tolerance mainly via targeted deacetylation of H3 and H4 histones. In *Oryza sativa*, OsHDA706 removes acetyl groups from H4K5 and H4K8 sites, which further represses the expression of *OsPP2C49*. Since *OsPP2C49* acts as a negative regulator of salt tolerance, the OsHDA706-mediated transcriptional inhibition ultimately improves rice salt adaptation [50]. In *Arabidopsis*, AtHDA9 catalyzes H3K9 deacetylation and suppresses more than 100 heat-responsive genes, highlighting its role in restraining excessive heat signaling activation [37]. Two negative drought regulators, SiHDA9 in *Setaria italica* and GhHDA8 in *Gossypium hirsutum*, also rely on H3K9 deacetylation to mediate drought responses [12,51]. Gene silencing of these two HDACs enhances plant drought tolerance, whereas their overexpression produces opposite phenotypes, which indicates that both proteins repress the transcription of positive drought-responsive genes. The MYB96-HDA15 module represses negative ABA regulators through H3 and H4 deacetylation, promoting drought resistance [42,54,55]. PtrHDA907/908 acts as a negative regulator of drought response in poplar by sustaining repressive histone deacetylation at the PtrECPP44 locus, a repression that is relieved during drought to restore oxidative homeostasis [53]. In banana, the MaMYB4-MaHDA2 protein complex suppresses the ω-3 fatty acid desaturase genes (*MaFADs*) by histone deacetylation at corresponding gene promoters. The inhibited transcription of *MaFAD* genes remodels membrane lipid composition and consequently improves banana cold adaptation [56].

As a plant-specific HDAC subfamily, HD2 proteins also regulate histone acetylation homeostasis to mediate abiotic stress. In *Arabidopsis*, AtHD2C interacts with AtHDA6 to co-deacetylate H3K9 and H3K14 [11]. The double mutant of these two genes shows elevated histone acetylation levels and sensitivity to ABA and salt stress, confirming that AtHD2C and AtHDA6 work synergistically to promote plant salt tolerance [11]. In tea plants, CsHD2C modulates H4 acetylation levels, which facilitates the binding of RNA polymerase II to promoters of stress-responsive genes. Heterologous overexpression of *CsHD2C* can restore the defective stress resistance phenotype of *Arabidopsis hd2c* mutant lines [30]. Moreover, AtHD2D positively regulates drought, salt, and cold stresses and promotes ROS accumulation in roots [57,58]. Given that enhanced ROS metabolism is recognized as a convergence point for stress signaling and root morphogenesis, we propose that AtHD2D contributes to stress adaptation primarily through modulating ROS homeostasis, which in turn facilitates lateral root development. Consistent functional patterns are also found in tomato and cotton. Tomato SlHDT1 positively regulates drought tolerance by decreasing histone acetylation levels. RNAi-mediated silencing of *SlHDT1* reduces chlorophyll and proline contents while aggravating membrane lipid peroxidation, thus disrupting cellular osmotic balance during drought stress [59]. Cotton GhHDT4D promotes drought tolerance through H3K9 deacetylation [52]. Nevertheless, HD2 members also carry out opposite regulatory functions. AtHDT4 acts as a negative drought regulator. Down-regulation expression of *AtHDT4* elevates H3K27ac levels at drought-response genomic loci and accordingly enhances drought resistance [43].

Compared with the above two subfamilies, the SIR2 subfamily has been less studied in plant abiotic stress regulation. AtSRT2 reduces H4K8 acetylation levels at the *AtVAMP714* locus, and this epigenetic modification modulates vesicle trafficking and affects plant salt tolerance [39].

In conclusion, histone lysine deacetylation mediated by HDACs is a central epigenetic mechanism that fine-tunes plant stress transcriptional responses. Each HDAC subfamily exhibits distinct selectivity for specific histone acetylation marks, such as H3K9, H3K14, H4K5, and H4K8. Such precise chromatin modifications enable plants to dynamically reprogram their genome-wide transcription profiles and to cope with diverse adverse environmental conditions.

### 3.3. Non-Histone Lysine Deacetylation Regulates Abiotic Stress

Histone tails represent only a fraction of substrates modified by plant HDACs. A broad spectrum of non-histone proteins also undergo lysine deacetylation mediated by these enzymes, ranging from transcription factors and central metabolic enzymes to cytoskeletal and ribosomal structural proteins (Table 3).

#### 3.3.1. Deacetylation of Stress Transcription Factors

In rice, OsHDA716 plays a negative regulatory role in drought and cold tolerance via deacetylation of the transcription factor bZIP46, which leads to decreased transcriptional activity and stability of the factor [63,64]. The opposite regulatory logic exists in apple plants. MdHDA6 improves plant cold hardiness by stripping acetyl groups off MdTCP15, and this deacetylated form of MdTCP15 exhibits stronger capacity to initiate transcription from cold-responsive promoters [13]. The multifunctional transcription factor AtWRKY53 orchestrates responses to an extensive suite of environmental challenges, spanning drought, high salinity, ozone, hydrogen peroxide and salicylic acid signaling, alongside defenses against invading pathogens [60,67,68,69,70]. AtHDA9 targets specific lysine residues on AtWRKY53 via deacetylation, and this post-translational modification ultimately renders Arabidopsis seedlings more susceptible to salt toxicity [60].

#### 3.3.2. Deacetylation of Metabolic and Redox Enzymes

Beyond transcription factors, metabolic enzymes serve as vital non-histone substrates for HDACs under oxidative and thermal stress. Rice OsSRT1, a SIR2 family NAD^+^-dependent deacetylase, has been shown to deacetylate glycolytic enzyme GAPDH when plants face oxidative damage [62]. Knockdown of *OsSRT1* elevates the cellular NADH/NAD^+^ ratio, while *OsSRT1* overexpression lowers this ratio. In line with such metabolic changes, RNAi-mediated suppression of *OsSRT1* leads to enhanced catalytic activity of GAPDH and additional glycolytic enzymes. This set of genetic evidence reveals a closed negative feedback circuit linking OsSRT1 function and cellular NADH/NAD^+^ redox balance. Beyond GAPDH, OsSRT1 also targets the non-histone protein OsPARP1, which plays a role in the DNA damage repair pathway, further linking NAD^+^-dependent deacetylation to oxidative stress management [71].

Whole-proteome profiling of heat-challenged rice reveals a global decline in protein deacetylation events, creating a demand for OsHDA714 to remodel key metabolic enzymes and maintain heat stress homeostasis [8]. Further research shows that OsHDA714 deacetylates stress granule proteins and ribosomal proteins, including RPS3, RPS6 and RPL7a [72]. Collectively, these results indicate that OsHDA714 responds to heat stress to deacetylate cellular proteins, control metabolic activities, stimulate stress granule formation, and confer heat tolerance in rice.

The connection between HDAC-mediated deacetylation and the glutathione-dependent ROS elimination system is exemplified by OsHDAC1. OsHDAC1-mediated deacetylation of OsALDH2B1 at K311 and K531 accelerates its degradation via the ubiquitin–proteasome system, an event that attenuates *OsGR3* transcription and, consequently, compromises glutathione-dependent ROS elimination [66]. In salt-treated *Arabidopsis*, AtHDA5 directly deacetylates GT2L and ERD7, two proteins controlling cellular H_2_O_2_ clearance [61]. In chrysanthemum, the sirtuin DgSRT2 enhances low-temperature tolerance through the ROS scavenging system, with overexpression lines showing higher antioxidant enzyme activities and lower H_2_O_2_ and superoxide accumulation [73]. Similarly, in *Arabidopsis*, the sirtuin AtSRT2 deacetylates non-histone proteins associated with mitochondrial energy metabolism and plays a role in osmotic stress responses [74]. Collectively, this post-translational cascade establishes a direct mechanistic link between histone deacetylase activity and the cellular redox buffering capacity.

Accumulating evidence highlights that HDACs act not only as epigenetic controllers of transcription but also as direct modulators of metabolic enzymes and antioxidant systems via non-histone lysine deacetylation. Notably, NAD^+^-dependent sirtuins in particular establish HDACs as core signaling hubs that interconnect carbon metabolism, cellular redox homeostasis and abiotic stress adaptation.

## 4. Mechanisms of HDACs in Abiotic Stress Responses

Plant histone deacetylases rely on an intricate multilayered regulatory network to coordinate stress responses, with lysine deacetylation on both histone and non-histone proteins serving as their core biochemical activity. This reversible modification reshapes surface charge and tertiary structure of target proteins, which in turn disturbs diverse cellular biochemical cascades. Collectively, these overlapping regulatory pathways enable HDACs to decode fluctuating environmental signals and calibrate transcriptomic reprogramming for survival under adverse growth conditions.

### 4.1. Regulation of Transcriptional Activity

HDACs modulate plant abiotic stress responses at the transcriptional level via two distinct regulatory pathways. The first pathway relies on histone deacetylation to remodel chromatin structure, while the second one targets transcription factors directly via non-histone deacetylation to manipulate their intrinsic transcriptional activity.

In the first chromatin-dependent pathway, HDACs inhibit target gene transcription universally through histone deacetylation. Histone acetylation neutralizes the positive charge of histone lysine residues, loosening chromatin structure and facilitating the binding of RNA polymerases and transcription factors to gene promoters. By contrast, HDACs remove acetyl groups from histone tails, restore electrostatic binding between histones and DNA, and condense chromatin into transcriptionally silent heterochromatin. Notably, the final regulatory effect on plant stress tolerance depends entirely on the function of inhibited target genes; on the one hand, multiple HDACs impair stress tolerance by suppressing positive stress-related genes. For example, foxtail millet SiHDA9 reduces H3K9 acetylation levels at the promoters of two drought-resistant positive genes, *SiRAB18* and *SiP5CS2*, repressing their expression and reducing drought tolerance of millet plants [51]. Similarly, cotton GhHDA8 and *Arabidopsis* AtSRT1 downregulate key positive stress regulators by reducing H3K9ac modification, thus suppressing plant drought and salt tolerance [75]. On the other hand, certain HDACs improve stress adaptability via inhibiting negative stress signaling genes. For example, the AtHD2C-AtHDA6 complex in *Arabidopsis* and GhHDT4D in cotton both positively regulate stress resistance by mediating the deacetylation of H3K9 to repress the transcription of ABA/drought negative regulators *ABI1*/*ABI2* and *GhWRKY33*, respectively, thereby activating downstream stress-responsive pathways [11,52].

Unlike chromatin-based transcriptional regulation, the second pathway enables HDACs to regulate stress signaling independent of chromatin structural changes. The functional substrates of this pathway are dominated by stress-related transcription factors. In this mode, HDACs directly deacetylate key transcription factors to alter their DNA-binding affinity, protein activity or subcellular localization and indirectly tone the transcription intensity of downstream stress-responsive genes without modifying the genome-wide histone acetylation landscape. A typical example is AtHDA9 in *Arabidopsis*. It directly interacts with transcription factor AtWRKY53 and deacetylates five specific lysine residues (K12, K26, K27, K58, K268) of AtWRKY53. Such deacetylation impairs the DNA-binding ability of AtWRKY53, restricts the expression of downstream stress defense genes, and finally increases plant sensitivity to salt stress [60]. Additional evidence from wheat further validates this model. TaHDA8 removes acetyl groups from transcription factor TaAREB3 to weaken its transcriptional activation capacity, forming a non-chromatin regulatory branch to fine-tune drought response intensity [65].

The current literature on plant HDACs under abiotic stress is heavily weighted towards histone substrates, a bias that stems from both technical convenience and conceptual legacy. Histone deacetylation can be readily probed using well-validated antibodies and chromatin immunoprecipitation assays, enabling genome-wide correlation with transcriptional outputs. Non-histone targets, by contrast, lack such generic tools; their identification depends largely on high-resolution mass spectrometry and subsequent biochemical validation for each candidate protein, a labor-intensive process that hampers large-scale discovery. Moreover, histone deacetylation leads to relatively predictable regulatory outcomes. It primarily represses gene expression through chromatin compaction. Non-histone acetylation, by contrast, produces diverse effects. These effects are often context-dependent. They can affect protein stability, enzymatic activity, or molecular interactions. Such variability makes it difficult to formulate generalizable models. These practical and conceptual asymmetries have historically guided research efforts towards the more tractable histone pathway. Nevertheless, the growing accessibility of proteomic platforms and the increasing recognition of non-histone acetylation as a critical node in stress signaling are progressively shifting this balance, and future investigations will likely uncover equally significant contributions from non-histone deacetylation events.

### 4.2. Regulation of Protein Stability

Protein turnover homeostasis becomes particularly vulnerable when plants face abiotic stress, and HDAC-driven deacetylation emerges as a vital switch controlling substrate degradation rates. Lysine acetylation frequently acts as a recognition tag for proteasome-mediated protein breakdown. Deacetylation, however, yields divergent outcomes on protein stability, and its final effect hinges on substrate identity and intracellular environmental context. In *Arabidopsis*, AtSRT1 physically interacts with MBP-1 and removes acetyl groups from specific lysine residues of MBP-1. Such deacetylation significantly stabilizes MBP-1 protein in vivo [75]. In rice, OsHDA714 deacetylates ribosomal proteins RPS3, RPS6 and RPL7a. Site-mimic assays verified that deacetylated variants of these ribosomal proteins display increasing stability, whereas acetylation-mimic mutations accelerate their degradation [72]. In contrast, OsHDA716 targets three lysine residues (K263, K281, and K294) on cold-responsive transcription factor bZIP46 and accelerates its proteolysis [64]. Taken together, HDAC-triggered lysine deacetylation can either stabilize or destabilize target functional proteins.

### 4.3. Regulation of Subcellular Localization

HDACs also reshape stress signaling by altering the subcellular trafficking of target functional proteins. Accumulating evidence indicates that acetylation-mediated regulation of protein subcellular localization is an evolutionarily conserved mechanism across plants and animals.

A typical case is the glycolytic enzyme GAPDH, whose acetylation-dependent nucleocytoplasmic shuttling represents a conserved regulatory mechanism across diverse organisms. In rice, OsGAPDH1 translocates into the nucleus to activate stress-related transcriptional cascades upon oxidative stress. OsSRT1, a representative SIR2-family deacetylase, eliminates acetyl groups from six lysine sites (K57, K74, K120, K217, K261 and K265) on GAPDH. This deacetylation modification inhibits GAPDH nuclear accumulation and represses its transcriptional activation capacity [62]. Notably, this regulatory mechanism is not restricted to rice. In *Arabidopsis*, cytosolic GAPDH undergoes acetylation under heat stress, which triggers its translocation to the nucleus, where it interacts with the transcription factor NF-YC10 to modulate the expression of heat-inducible genes [76]. Beyond GAPDH, lysine acetylation has also been proven to modulate protein subcellular localization in WRKY63 [77]. In *Arabidopsis*, the group-III WRKY transcription factor WRKY63 undergoes acetylation at lysine 31 (K31). This acetylation is antagonistically regulated by the histone acetyltransferase HAG1 and the histone deacetylase HDA6. Specifically, HAG1-mediated acetylation enhances the nuclear localization of WRKY63, whereas HDA6-mediated deacetylation reduces its nuclear retention and thereby represses WRKY63-mediated transcriptional activation.

In mammalian systems, analogous mechanisms have also been documented. The m6A methyltransferase METTL3 undergoes acetylation at K177 by p300, which weakens its nuclear import and retains it in the cytoplasm, while SIRT1-mediated deacetylation promotes its nuclear translocation [78]. The autophagy-related protein LC3 undergoes acetylation that triggers its translocation from the nucleus to the cytoplasm to initiate autophagy [79]. Hyperacetylation of HMGB1 nuclear localization signal (NLS) sequence promotes its release from the nucleus to the cytoplasm [80].

Studies from plants and animals draw consistent conclusions. Reversible acetylation and deacetylation alter protein subcellular localization. This regulatory mode is highly conserved in eukaryotes and shapes the biological functions of target proteins.

### 4.4. Crosstalk Between Deacetylation and Methylation Under Abiotic Stress

Beyond the independent regulatory roles of HDACs on histone and non-histone protein deacetylation, HDACs coordinate dynamically with histone methylation to orchestrate plant epigenetic landscapes during abiotic stress adaptation, forming interlocked epigenetic regulatory circuits rather than isolated modification pathways. In *Arabidopsis*, HDA6 interacts with FLD to cooperatively erase acetylation and methylation marks at stress-sensitive loci, thereby reinforcing transcriptional repression [81]. Notably, disruption of HDA6 function often perturbs the deposition of histone methylation marks, indicating that HDAC activity is a prerequisite for balanced methylation homeostasis [82]. In rice, histone demethylase JMJ704 forms a chromatin modification complex with H3K9ac-specific deacetylase HDA709. Under salt stress, the complex is recruited by the transcription factor WRKY72 to the promoter regions of peroxidase genes and salt-responsive genes, achieving transcriptional silencing of the target gene by simultaneously removing H3K4me3 and H3K9ac activation modifications [83]. Despite these insights, most evidence remains limited to pairwise interactions between individual HDACs and single methylation modifiers under isolated stress conditions; the tissue-specific and temporal dynamics of such crosstalk, as well as possible subfamily-specific divergence between RPD3, SIR2 and HD2 members across species, await systematic exploration.

### 4.5. Redox-Dependent Regulation of HDAC Activity

Beyond their roles in deacetylating histones and non-histone proteins to modulate stress responses, HDACs themselves are subject to regulation by the cellular redox state. This establishes a bidirectional regulatory loop in which HDACs both respond to and shape the oxidative environment under abiotic stress.

Reactive oxygen species (ROS) and reactive nitrogen species (RNS) can directly modify HDAC proteins through oxidative post-translational modifications, thereby altering their enzymatic activity, subcellular localization, and substrate accessibility. For instance, *Arabidopsis* HDA19 undergoes S-nitrosylation at Cys137 under oxidative stress, promoting its nuclear enrichment, enhancing deacetylase activity, and repressing stress-responsive genes to improve stress tolerance. This modification depends on stress-elevated NO levels, positioning HDA19 as a redox sensor linking oxidative signals to chromatin regulation [84].

For the SIR2/sirtuins class, the regulatory connection to redox state is even more direct, as their deacetylase activity is strictly dependent on NAD^+^ as a substrate. The intracellular NAD^+^ concentration and the NAD^+^/NADH ratio are major determinants of sirtuin catalytic efficiency. Oxidative stress alters the NAD^+^/NADH ratio, directly impacting OsSRT1 function. Knockdown of *OsSRT1* leads to oxidative burst and cell death, whereas *OsSRT1* overexpression enhances tolerance to oxidative stress via transcriptional reprogramming of redox-related genes [62].

Collectively, these findings reveal a bidirectional regulatory loop. ROS/RNS modify HDAC activity through oxidative post-translational modifications and alter NAD^+^ availability to modulate sirtuin function; in turn, HDACs regulate the expression and activity of antioxidant enzymes and metabolic enzymes, shaping the cellular redox landscape. This redox–epigenetic coupling ensures that chromatin and protein acetylation dynamics are continuously adjusted to the prevailing oxidative status of the cell, enabling rapid and appropriate stress acclimation.

### 4.6. Summary of HDAC Regulatory Mechanisms Under Abiotic Stress

Notably, HDACs exhibit contrasting regulatory roles in plant stress responses, with individual members either promoting or repressing defense behaviors. This functional duality cannot be attributed to a single determinant but arises from the synergistic integration of multiple internal and external cues, including substrate properties, tissue-specific microenvironments, stress types, plant developmental stages, and endogenous phytohormone signaling. Substrate identity acts as the most direct determinant of HDAC functional output. HDAC-mediated deacetylation can stabilize, activate, or degrade target proteins, and the ultimate stress response is fundamentally determined by whether the modified substrate serves as a positive or negative component in local stress signaling pathways. Tissue and organ specificity further constrains the spatial distribution of HDAC proteins and their available substrates, establishing distinct context-dependent regulatory patterns. Different abiotic stresses, including drought, salinity, and extreme temperature, also activate divergent upstream signaling cascades that differentially tune HDAC activity and downstream target selection. In addition, plant developmental status resets the physiological background throughout growth, dynamically balancing stress defense activation and normal metabolic growth. Critically, stress-responsive phytohormones, including ABA, JA, and SA, act as central signal integrators that connect fluctuating external environmental stimuli with internal HDAC-mediated acetylation regulatory machinery. These multilayered regulatory factors function cooperatively rather than independently, enabling plants to precisely and spatiotemporally fine-tune stress adaptation according to specific internal and external conditions.

**Figure 2:**
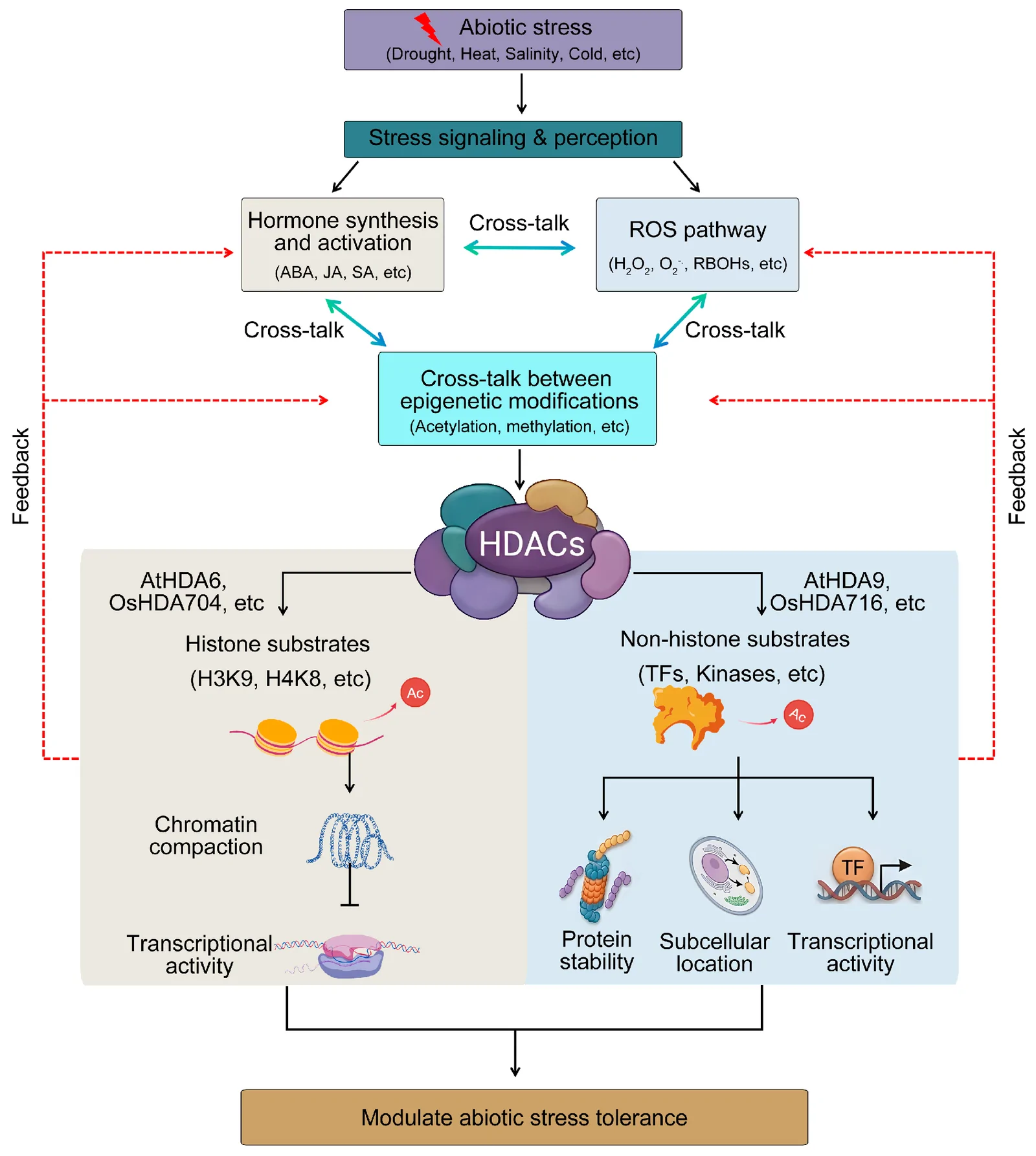
Schematic model of HDACs regulating abiotic stress in plants. Upon the perception of abiotic stress signals, plants activate ROS and phytohormone signaling pathways. Mutual crosstalk exists between these two pathways, and both interact with epigenetic regulatory networks. As a form of epigenetic modification, HDAC-catalyzed deacetylation of histone and non-histone substrates modulates transcriptional activity, target protein stability and subcellular localization, thereby ultimately governing plant tolerance to abiotic stress. Meanwhile, HDACs-dependent signaling pathways exert feedback regulation on ROS signaling, phytohormone cues and epigenetic modification events. Bidirectional arrows denote crosstalk between two components, while red dashed arrows indicate feedback regulation.

Taken together, HDACs participate in plant abiotic stress adaptation through a combination of transcriptional and post-translational epigenetic modulation (Figure 2). At the chromatin level, HDACs mainly mediate transcriptional repression via histone deacetylation, which compacts chromatin structure. HDACs are also capable of targeting transcription factors directly to change their activity, which affects stress-related gene expression without altering chromatin configuration. Beyond transcriptional control, HDACs also exert post-translational regulation by modulating the stability and intracellular localization of key functional proteins. HDACs are modulated by upstream phytohormone and reactive oxygen species (ROS) signals. Meanwhile, downstream signals regulated by HDACs exert feedback control over ROS and phytohormone signaling. Moreover, extensive crosstalk occurs among different epigenetic modifications. In summary, HDACs modulate abiotic stress responses via sophisticated signaling networks.

## 5. Conclusion and Future Perspectives

### 5.1. Comprehensive Summary of HDAC-Mediated Abiotic Stress

This study systematically illustrated the classification, structural characteristics, and conserved catalytic mechanisms of three plant HDAC subfamilies, namely RPD3/HDA1, SIR2 and plant-specific HD2. We further generalized their divergent functional characteristics in abiotic stress regulation from the dimensions of stress response spectrum, substrate preference and overall regulatory outcome.

The widely distributed RPD3/HDA1 subfamily acts as the core epigenetic regulator across nearly all types of abiotic stresses. Its members can deacetylate both histone and non-histone substrates at comparable frequencies. SIR2-type HDACs also function in various abiotic stresses. Distinct from the other two subfamilies, plant-unique HD2 proteins are mainly involved in drought and ABA signaling. All three clades contain isoforms with bidirectional regulatory capacity for abiotic stress, yet their distinct stress spectra and substrate selectivity create clear functional divergence. Beyond independent subfamily traits, the three HDAC lineages form a coordinated regulatory system with clear division of labor and mutual crosstalk under abiotic stress. Under single or combined adverse stimuli, the three subfamilies activate synergistic molecular routines to jointly reshape plant transcriptomes, sharing several conserved core regulatory patterns. First, all HDAC proteins rely on lysine deacetylation as the core catalytic activity to repress the transcriptional activity of bound target molecules, and their final stress-regulating polarity is uniformly governed by the functional attributes of downstream substrates. Second, every subfamily coordinates chromatin-dependent histone modification and chromatin-independent transcription factor regulation to calibrate stress signal strength, even though they differ in preferred substrate types. Third, HDACs from all three clades establish bidirectional feedback loops with upstream hormone signals and other epigenetic modifiers (DNA and histone methyltransferases), dynamically adjusting repression intensity to avoid excessive or insufficient stress response. Such labor division and shared molecular logic jointly construct a complete multi-layer epigenetic network centered on plant HDACs, rather than three isolated independent regulatory modules. To systematically clarify this bidirectional regulatory feature, we established unified molecular criteria to judge whether an HDAC exerts positive or negative effects on plant stress tolerance. Biochemically, HDACs inherently suppress the transcriptional activity of bound target molecules by eliminating lysine acetylation; their final regulatory polarity is entirely dictated by the biological function of downstream substrates. HDACs function as negative stress regulators when they erase acetyl marks from histones at promoters of stress-positive genes or inactivate stress-activating transcription factors, such as AtHDA9 and AtSRT1, which jointly repress drought and salt defense signaling via dual histone and transcription factor targeting. By contrast, HDACs serve as positive regulators of stress resistance if their substrates are stress-inhibitory repressors. For instance, the AtHD2C–AtHDA6 complex and cotton GhHDT4D deacetylate histones at loci encoding ABA-negative regulators, blocking repressive signal cascades and activating plant drought tolerance. Three core interrelated molecular factors determine such divergent HDAC functional orientations: substrate recognition specificity acts as the decisive basis, stress- and tissue-specific expression patterns provide contextual functional switches, and synergistic or antagonistic crosstalk with DNA/histone methylation modifiers further modulates the intensity of HDAC-mediated transcriptional repression. We attribute this disparity mainly to the larger gene copy number of RPD3/HDA1, which grants this subfamily versatile substrate recognition capability. Meanwhile, abundant stress-responsive *cis*-elements in their promoters and intricate protein interaction networks further expand their regulatory range across diverse stress environments.

The results further clarified how HDACs mediate plant responses to multiple abiotic stresses through multi-layer regulatory mechanisms at transcriptional and post-translational levels: upstream phytohormone signaling triggers HDAC transcription, followed by HDAC-mediated histone deacetylation remodeling chromatin status and non-histone protein deacetylation modulating target protein activity, while HDACs dynamically crosstalk with DNA methylation and histone methylation to shape stress-responsive epigenetic landscapes. Different HDAC members exert either positive or negative effects on abiotic stress resistance depending on their downstream target genes, jointly forming a complicated but orderly epigenetic regulatory network for plant stress adaptation.

In contrast to previous review papers on plant HDACs, which mostly focus on isolated HDAC biochemical properties or only summarize HDAC functions under single abiotic stress types, this review establishes three unique integrative frameworks that fill obvious gaps in the existing literature and highlight its differentiated academic value. First, earlier reviews separately discuss histone modification regulation mediated by HDACs without constructing a complete linear regulatory cascade covering environmental stimuli to plant stress phenotypes. This work integrates upstream hormone signal transduction, dual downstream substrate regulatory branches, and epigenetic crosstalk networks and optimizes the schematic diagram to visualize the full stress response pathway, realizing systematic integration of the whole regulatory chain. Second, nearly all prior HDAC reviews put forward generalized, unified research prospects without distinguishing the divergent research demands of model plants and economic crops. This paper divides targeted research directions for basic theoretical exploration and crop breeding application, respectively, and proposes matched technical routes according to the distinct research bottlenecks of two plant types. Third, existing summaries only briefly mention the interaction between HDACs and other epigenetic modifications with shallow mechanistic analysis. We systematically sort out the synergistic and antagonistic regulatory relationships between HDAC deacetylation, histone methylation and DNA methylation under diverse abiotic stresses and elaborate bidirectional feedback loops among epigenetic modifiers, greatly deepening the understanding of complex epigenetic cross-regulation compared with previous reviews.

### 5.2. Research Limitations of HDAC-Mediated Abiotic Stress

Although current research has uncovered core functions of HDACs in plant stress adaptation, several prominent research gaps still restrict the in-depth application of HDAC epigenetic regulation in crop stress resistance breeding.

First of all, existing HDAC studies exhibit obvious research bias and priority differences between model plants and economic crops. Most mechanistic studies have been comprehensively conducted in model plants (such as *Arabidopsis* and rice), where the conserved stress-responsive functions, upstream regulatory pathways and downstream target networks of HDAC family members have been preliminarily clarified. In contrast, research on horticultural and economic crops (including fruits, vegetables, and industrial crops) is still poorly understood. The species-specific functional differentiation of HDACs, the epigenetic regulatory specificity responding to unique abiotic stresses, and the elite allele resources of HDACs in economic crops are still poorly explored.

Second, the crosstalk between HDAC-mediated deacetylation and other epigenetic modifications (such as DNA methylation and histone methylation) during stress responses requires further exploration. Most existing studies merely report simple protein interactions between individual HDAC isoforms and single methylation-regulatory enzymes, lacking systematic multi-omics tracing of dynamic epigenetic crosstalk trajectories throughout the whole stress-response process. Key unresolved questions include how different HDAC subfamilies selectively coordinate with distinct histone methyltransferases, demethylases and DNA methyltransferases under abiotic stresses, as well as whether crop-specific heterochromatin characteristics reshape the HDAC-methylation regulatory cascade. At present, few investigations have tracked the sequential order of acetylation and methylation modification deposition at stress gene loci, and the upstream signal triggers that switch the synergistic or antagonistic relationships between these two epigenetic pathways are largely unknown.

Third, most studies stay at the laboratory functional verification stage, and few HDAC-based epigenetic breeding materials have been applied in field production.

### 5.3. Targeted Future Research Directions

Importantly, future research priorities must be clearly differentiated between model plants and economic crops, as their research objectives and technical constraints are fundamentally distinct.

For model plants, the priority lies in mechanistic decoupling and system-level reconstruction of HDAC regulatory networks. Given the widespread functional redundancy and pleiotropic effects among different HDAC family members, future studies should concentrate on resolving the spatiotemporal dynamics of HDAC complexes in native chromatin environments with the assistance of advanced imaging and proximity-labeling techniques. Multi-omics integration strategies, including time-series ChIP-seq, ATAC-seq, and RNA-seq under various abiotic stress treatments, can further help dissect the combinatorial histone modification codes that govern stress-responsive gene transcription. In addition, synthetic biology-based reconstitution of minimal HDAC regulatory modules is essential to verify the sufficiency and necessity of HDAC-mediated epigenetic regulation in plant stress adaptation, laying a solid theoretical foundation for subsequent applied research in crops.

For economic crops, the research priority shifts toward application-oriented precision breeding and field resilience improvement. Current economic crop research is severely restricted by large and complex genomes, widespread genetic redundancy, and the common yield–stress-tolerance trade-off effect. To address these bottlenecks, future efforts should focus on exploring superior natural HDAC allelic variations from core crop germplasm resources via haplotype-based GWAS and CRISPR-Cas9-mediated allele swapping, specifically screening elite alleles that confer robust stress tolerance without sacrificing crop yield. Moreover, developing stress-inducible and tissue-specific synthetic promoters enables precise manipulation of HDAC expression, which effectively relieves the adverse pleiotropic effects on vegetative growth while enhancing stress protection in key reproductive tissues. Furthermore, stacking multi-gene editing modules that combine HDACs with downstream regulatory factors can synergistically optimize stress response networks, and multi-location field trials are required to validate the practical adaptability and yield performance of edited materials under complex field climate conditions. Collectively, the differentiated and in-depth exploration of HDAC epigenetic functions in different types of plants will promote the innovative development of crop epigenetic breeding and offer reliable theoretical and technical solutions to breed high-yield and stress-resilient crops under increasingly severe global climate changes.

## Figures and Tables

**Table 1:** HDAC genes of different plant species.

Species	Gene Number	Subfamily			References
		RPD3/HDA1	SIR2	HD2	
*Capsicum annuum*	15	10	2	3	[18]
*Gossypium hirsutum*	29	18	4	7	[19]
*Gossypium arboretum*	15	9	2	4	[19]
*Gossypium raimondii*	13	9	2	2	[19]
*Triticum aestivum*	49	36	6	7	[16]
*Oryza sativa*	17	14	2	1	[20]
*Brachypodium distachyon*	14	11	2	1	[20]
*Hordeum vulgare*	15	13	2	0	[20]
*Oryza rufipogon*	14	12	1	1	[20]
*Sorghum bicolor*	19	12	2	5	[21]
*Setaria italica*	15	12	2	1	[20]
*Zea mays*	19	12	2	5	[20]
*Prunus trichocarpa*	15	10	3	2	[22]
*Prunus mume*	13	9	2	2	[22]
*Prunus armeniaca*	12	9	1	2	[22]
*Prunus salicina*	13	9	2	2	[22]
*Prunus persica*	13	9	2	2	[22]
*Prunus avium*	16	12	2	2	[22]
*Prunus yedoensis*	13	11	2	0	[22]
*Rosa chinensis*	20	13	4	3	[22]
*Fagopyrum tataricum*	14	9	2	3	[23]
*Populus trichocarpa*	16	11	2	3	[24]
*Vitis vinifera*	13	10	2	1	[25]
*Salvia miltiorrhiza*	16	12	2	2	[26]
*Phyla nodiflora*	17	11	2	4	[27]
*Litchi chinensis*	11	9	2	0	[28]
*Cannabis sativa*	14	10	2	2	[29]
*Camellia sinensis*	18	8	4	6	[30]
*Dendrobium officinale*	14	10	2	2	[31]
*Arabidopsis thaliana*	18	12	2	4	[32]
*Glycine max*	28	18	4	6	[33]
*Hibiscus cannabinus*	6	5	1	0	[17]
*Raphanus sativus*	21	13	2	6	[34]

**Table 2:** HDAC-mediated histone deacetylation in abiotic stress regulation.

HDACs	Species	Histone Targets	Target of Histones	Abiotic Stress	Regulatory Effect	References
AtHD2C	*A. thaliana*	H3K9/K14	*ABI1* and *ABI2*	ABA *	Positive	[11,40]
AtHDA6	*A. thaliana*	H3K9/K14	*ABI1* and *ABI2*	ABA * and salt	Positive	[11,41]
AtHDA15	*A. thaliana*	H3, H4	*ROP6*, *ROP10*, and *ROP11*	ABA *	Positive	[42]
AtHDT4	*A. thaliana*	H3K27	—	Drought	Negative	[43]
AtSRT1	*A. thaliana*	H3K9	*LOS2*/*ENO2* and *STZ*/*ZAT10*	ABA *, salt and osmotic	Negative	[44]
AtSRT2	*A. thaliana*	H4K8	*VAMP714*	Salt	Positive	[39]
AtHDA9	*A. thaliana*	H3K9	*WRKY33*	Heat	Positive	[37]
AtHDA9	*A. thaliana*	H3K9	*LIP4*, *LTP6*, *LIP3*, *PAD3*, *GST1*, *RAP2.6*, *MYB29*, *CYP79B2*, *GOLS2*, *PLC1*, *IMS3*, *ANN1*, *AAP6*, *GSTF10* and *ANN4*	Salt and drought	Positive	[45]
OsHDA704	*O. sativa*	H3, H4K5/K12	*DST* and *ABIL2*	Salt and drought	Positive	[46]
OsHDA704	*O. sativa*	H4K5/K8	*ABI5*	Drought	Positive	[47]
OsHDA710/OsHDAC2	*O. sativa*	H3, H4K5 and H4K16	*LEA3*, *bZIP72*, *NHX1 72* and *ABI5*	Salt and drought	Negative	[48]
OsHDA1	*O. sativa*	H3	*LEA1* and *SOS1*	Salt	Negative	[49]
OsHDA706	*O. sativa*	H4K5/K8	*PP2C49*	Salt	Positive	[50]
SiHDA9	*S. italica*	H3K9	*RAB18*, *RAP2.4*, *P5CS2*, *RD22*, *PIP4-1*, and *LHCB2.3*	Dehydration	Negative	[51]
GhHDT4D	*G. hirsutum*	H3K9	*WRKY33*	Drought	Positive	[52]
PtrHDA907/908	*Populus* *tomentosa*	H3K9/K14/K27	*ECPP44*	Drought	Negative	[53]

* Abscisic acid.

**Table 3:** HDAC-mediated non-histone deacetylation in abiotic stress regulation.

HDACs	Species	Target Proteins	Target Sites	Abiotic Stress	Regulatory Effect	Reference
AtHDA9	*A. thaliana*	WRKY53	K12, K26, K27, K58, K268	Salt	Negative	[60]
AtHDA5	*A. thaliana*	GT2L and ERD7	—	Salt	Negative	[61]
AtSRT1	*A. thaliana*	MBP1	—	ABA, salt and osmotic	Negative	[44]
OsSRT1/OsSRT701	*O. sativa*	GAPDH	K57, K74, K120, K217, K261 and K265	Oxidative stress	Positive	[62]
OsHDA716	*O. sativa*	bZIP46	K263, K281 and K294	Drought	Negative	[63]
OsHDA716	*O. sativa*	bZIP46	K263, K281 and K294	Cold	Negative	[64]
OsHDA714	*O. sativa*	SG, RPS3, RPS6 and RPL7a	—	Heat	Positive	[8]
TaHDA8	*Triticum* *aestivum*	AREB3	K248 and K281	Drought	Negative	[65]
OsHDAC1	*O. sativa*	ALDH2B1	K311 and K531	Salt	Negative	[66]

## Data Availability

No new data were created or analyzed in this study. Data sharing is not applicable to this article.
